# Exploring physicochemical property and food safety in dried shrimp production: Risks and mitigation strategies

**DOI:** 10.1371/journal.pone.0342315

**Published:** 2026-02-09

**Authors:** Fauziah Fitriana, Saran Anuntawirun, Nawaphorn Roongrojmongkhon, Piyachat Dachsri, Edward R. Atwill, Anyarat Thiptara, Saharuetai Jeamsripong

**Affiliations:** 1 Department of Veterinary Public Health, Faculty of Veterinary Science, Chulalongkorn University, Bangkok, Thailand; 2 Department of Fisheries, Surat Thani Coastal Aquaculture Research and Development Center, Kanchanadit, Surat Thani, Thailand; 3 Department of Population Health and Reproduction, School of Veterinary Medicine, University of California, Davis, California, United States of America; 4 Veterinary Research and Development Center (Upper Southern Region), Thung-Song, Nakhon-Si-Thammarat, Thailand; 5 Research Unit in Microbial Food Safety and Antimicrobial Resistance, Department of Veterinary Public Health, Faculty of Veterinary Science, Chulalongkorn University, Bangkok, Thailand; Prince of Songkla University, THAILAND

## Abstract

Dried shrimp is a widely consumed aquatic product whose safety and quality depend on various physicochemical and microbiological properties influenced by production practices. This study aimed to assess the physicochemical characteristics and levels of bacterial contamination occurring in small-scale dried shrimp production and to propose practical mitigation strategies. Samples of dried shrimp were collected from Nakhon Si Thammarat and Surat Thani, Thailand and analyzed for moisture content, water activity, pH, salt concentration, and bacterial contamination; in addition, we collected information regarding each farmer’s production practices. The main shrimp species were *Parapenaeus longipes* and *Acetes* spp. Only 52.0% of samples met the moisture content standard. Production practices, particularly post-cooking handling, were identified as critical points for re-contamination and/or regrowth. While cooking reduced bacterial loads, subsequent handling stages led to significant increases in bacterial loads, emphasizing the need for improved hygiene during post-cooking handling. Average reported measurements from all shrimp species were salt (7.8 ± 5.3 g/100g), moisture (31.2 ± 11.1 g/100g), water activity (0.8 ± 0.09), and pH (7.9 ± 0.33). High overall prevalence of total coliforms (94.0%), fecal coliforms (41.3%), and *Escherichia coli* (26.4%) were found, with exceedances of standard limits at 71.6%, 19.4%, and 26.4%, respectively. *Salmonella enterica* subsp. *enterica* (1.0%; n = 2/201), *Staphylococcus aureus* (2.5%; n = 5/201), and *Vibrio cholerae* (2.0%; n = 4/201) were identified at low frequencies. The serovars of *Salmonella* were Tallahassee and Mount Pleasant. The mean microbial load of *S. aureus* samples was 4.2 logCFU/g, and all these samples exceeded the microbiological limit of 100 CFU/g set by the Thai Agricultural Standard. Negative binomial regression identified pH, sampling location, shrimp color, and fecal coliforms as predictors of *E. coli* levels. This study highlights the importance of microbial safety and quality assessment in dried shrimp production, emphasizing the need for stringent hygiene controls and critical control point management to prevent post-processing contamination and to ensure high product quality.

## Introduction

Growth in aquaculture of seafood commodities is being driven by rising global demand for these commodities, with Thailand being a major producer and exporter of seafood commodities. In 2019, approximately 60% of the world’s seafood came from aquaculture, and Thailand’s shrimp production nearly doubled from 33,051 MT in 2017–60,954 MT in 2019 [1]. Ensuring the safety of dried seafood, where drying serves as the main preservation method, remains a major challenge in several Asian countries given that previous studies have reported contamination with *Escherichia coli*, *Staphylococcus aureus*, and *Vibrio* spp of dried seafood [2–4]. For example, a foodborne outbreak in Singapore was linked to dried anchovies contaminated with multidrug-resistant *Salmonella* Typhimurium [5].

Dried shrimp, valued for its flavor and long shelf life, is commonly produced through traditional small-scale methods that are prone to microbial contamination at various stages of production, from fishing to processing to packaging [6]. In Southeast Asia, *Acetes* spp. are frequently processed into dried or fermented products using artisanal methods [7]. *Litopenaeus vannamei,* commonly found in large quantities in the Gulf of Thailand and the Andaman Sea, is often used for dried shrimp production. The preferred shrimp varieties can vary depending on the production location and consumer preferences [8]. Heating during the drying stage plays a key role in decontamination, but subsequent steps such as salting or handling may inadvertently reintroduce pathogens. Each stage of production, including washing, cooking, drying, and packaging, presents contamination risk, highlighting the importance of proper hygiene and process control [7].

Dried seafood is at substantial risk of contamination from *Escherichia coli*, *Staphylococcus aureus*, *Salmonella* spp.*, Vibrio parahaemolyticus*, and *V. cholerae* [2]. *E. coli* serves as a key sanitation indicator, making it a proxy for the quantitative assessment of seafood safety. Controlling water activity is critical measure to limit pathogen proliferation in ready-to-eat seafood products [9]. Several studies have explored microbial contamination in both raw and processed forms [2,10–12]. *E. coli*, *S. aureus*, and *Salmonella* spp*.* are specifically prioritized under the Thai Agricultural Standard (TAS) for microbiological safety in dried shrimp [13]. Although *V. cholerae* is not listed in the TAS, we included this pathogen in this study due to its recognized seafood-borne transmission risk and public health importance.

There remains a notable gap in comprehensive research focusing on indicator and pathogenic bacterial contamination throughout the entire production process of dried shrimp. This study aims to characterize the contamination dynamics of dried shrimp from pre- to post-processing stages by (1) quantifying pre-processing baseline contamination of total coliforms, fecal coliforms, and *E. coli*; (2) evaluating reductions in contamination following the cooking or heating process; and (3) identifying evidence of post-processing re-contamination and associated risk factors, including the presence of *S. aureus*, *Salmonella* spp., and *V. cholerae*. The findings from this study provide valuable insights for microbial contamination risk assessment and support the development of targeted interventions to enhance seafood safety throughout the production of dried shrimp products.

## Materials and methods

### Study questionnaire related to the production of dried shrimp

The cross-sectional study was conducted between September 2024 and January 2025 from two major shrimp-producing provinces in Thailand, Nakhon Si Thammarat and Surat Thani, as identified by the Department of Fisheries. All participants provided written informed consent before completing the questionnaire. Using in-person interviews, the survey data was collected from 12 out of 60 households (20%) involved in dried shrimp production. These household shrimp farmers were selected based on their willingness to participate in the study and the feasibility of sampling throughout the shrimp drying process. While this selection of household farmers was non-random, this sample size and proportion of our sample population was considered sufficient to capture the diversity of production practices among small-scale dried shrimp farmers in the study area.

Household participants were selected based on the following criteria: aged 18 years or older, at least three years of experience in dried shrimp production, and demonstrated their ability to communicate in Thai via phone or the internet. Individuals with less experience or communication limitations were excluded.

The questionnaire covered key aspects of smaller-scale dried shrimp production process, including location of dried shrimp production, the year(s) of experience of dried shrimp processing, season and frequency of production (e.g., winter, summer, rainy season, or all seasons), method of catching raw fresh shrimp, species and size of shrimp being targeted (e.g., black tiger shrimp (*Penaeus monodon*), Pacific white shrimp (*P. vannamei*), or other types), method of sizing and cooking shrimp, addition of salt, food coloring, duration of drying shrimp, option of peeling, storage conditions, duration before distribution, and monthly volume of dried shrimp production.

### Sample collection

**Fig 1:**
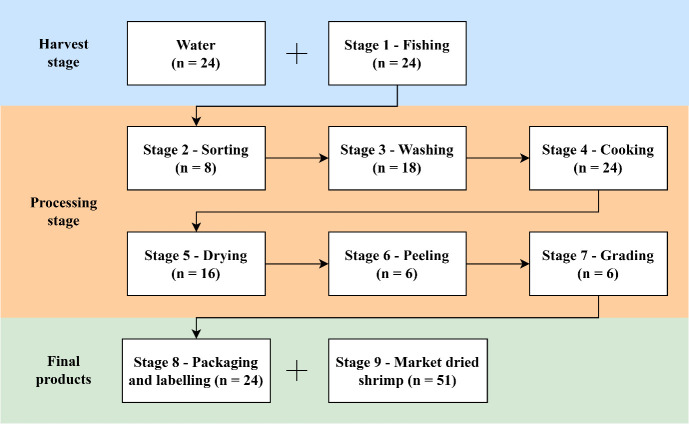
Diagram of sample collection and dried shrimp processing (n = 201): water samples (n = 24), processing samples (n = 102), and final product samples (n = 75).

A total of 201 samples were collected between December 2023 and February 2025, including 24 water samples, 102 processing samples, and 75 final dried shrimp product samples. During the harvest stage, samples of both water (n = 24) and shrimp (n = 24 from Stage 1 – fishing) were collected. Shrimp samples were obtained throughout the processing stages, which were classified as stages 2–7: stage 2 – sorting (n = 8), stage 3 – washing (n = 18), stage 4 – cooking (n = 24), stage 5 – drying (n = 16), stage 6 – peeling (n = 6), and stage 7 – grading (n = 6) (Fig 1). For the final products ready for consumption (n = 75), samples were taken from stage 8 – packaging and labelling (n = 24) and also from retail sources (n = 51). Some stages may have been omitted depending on the type of shrimp and the processing methods used by that particular household. To account for potential seasonal variations, we collected samples throughout Thailand’s three major seasons, as defined by the Thai Meteorological Department: winter (n = 26), summer (n = 62), and rainy (n = 113). This methodology of sampling across the various processing stages resulted in a total of 201 samples being collected from 12 consenting and accessible household farmers located throughout the Nakhon Si Thammarat and Surat Thani provinces, along with market-dried shrimp from retail stores from five different provinces: Bangkok, Nonthaburi, Samut Sakhon, Suphan Buri, and Trang provinces, based on store availability. These 201 samples were then used to calculate the prevalence of microbial contamination of dried shrimp across the various stages of processing, while also capturing the breadth of microbial diversity across different operational scales and management styles representative of the dried shrimp farming community in this region of Thailand. The number of samples collected for each season, along with the sample types and processing stages, is summarized in the supplementary data.

A total of 24 water samples were collected from shrimp-processing sites in Tha Sala District, Nakhon Si Thammarat (n = 8), Kanchanadit District, Surat Thani (n = 8), and Mueang District, Surat Thani (n = 8). The standard method for the examination of water and wastewater was used [14]. Two to three hundred ml of water from a depth of 40–60 cm below the surface were collected into sterile polypropylene bottles at the same time and location where the fresh shrimp samples were being collected, which helped ensure that the water samples were representative of the environment where the shrimp were harvested.

A minimum of 300 grams per shrimp sample were collected using sterile gloves for both post-harvest and retail market dried shrimp. All samples were maintained <10°C on ice in a sealed cooler and processed within 24 hours at the Department of Veterinary Public Health, Faculty of Veterinary Science, Chulalongkorn University, Bangkok, Thailand.

### Environmental parameters

The environmental parameters were recorded at the various harvest sites. The average and standard deviation (S. D.) of wind speed (m/s), maximum wind speed (m/s), relative humidity (%), and ambient air temperature (°C) were recorded during the collection of harvested fresh shrimp using a Kestrel 3000 Weather Meter (Nielsen-Kellerman, Bothwyn, PA, USA). In addition, the local meteorological data, including dry temperature (°C), maximum temperature (°C), minimum temperature (°C), 24-hour cumulative rainfall (mm), relative humidity (%), and wind speed (Knots/hr) were obtained from the Thai Meteorological Department (www.tmd.go.th).

### Detection and confirmation of bacteria

#### Determination of total viable count.

The total viable counts were obtained using standard methods for aerobic plate count as described in the United States Food and Drug Administration Bacteriological Analytical Manual (U.S. FDA BAM) [15]. Briefly, approximately 25 grams of shrimp or 25 ml of water were added to 225 ml of buffered peptone water (BPW) (Difco, Becton Dickinson, MD, USA). The mixture was then serially diluted tenfold, one ml of each dilution transferred into sterile petri dishes, and Molten Plate Count agar (PCA) (Difco) then poured over the sample and mixed thoroughly. Plates were incubated at 37°C for 24–48 hours and the number of colonies reported as colony-forming units per gram (CFU/g for shrimp and CFU/ml for water) of sample.

#### Enumeration and quantification of total coliforms, fecal coliforms, and *E. coli.*

Total coliforms, fecal coliforms, and *E. coli* were enumerated using a 3-tube most probable number (MPN) standard protocol from the U.S. FDA BAM [16]. In triplicate, one ml of each BPW dilution from prepared sample suspension were inoculated into 9 ml of lactose broth (Difco) containing a Durham tube. The samples were then incubated overnight at 37°C, and if gas production was observed, the tube was considered presumptive for total coliforms. These positive tubes are then transferred to Brilliant Green Lactose Bile (BGLB) broth and incubated under the same conditions to confirm as total coliform. One loopful of incubated lactose broth suspension was transferred to EC broth (Difco) and incubated at 44.5°C for 24–48 hours. The MPN for total and fecal coliforms was determined based on the number of gas-positive tubes observed in the BGLB and EC broth.

In addition, one loopful of the positive EC broth was streaked onto Levine’s eosin-methylene blue (L-EMB) (Difco) and MacConkey agar (Difco) agar plates. The plates were incubated at 37°C for 24 hours. Presumptive colonies of *E. coli* showed green metallic sheen on L-EMB agar and pink colonies on MacConkey agar. Suspected colonies of *E. coli* were Gram stained and biochemically confirmed using indole, triple sugar iron (TSI), citrate, catalase, and oxidase (Difco) tests. *E. coli* ATCC 25922 was used as a positive control. The concentration of *E. coli* (MPN/g) was calculated based on the number of positive EC tubes that exhibited confirmed *E. coli* colonies.

#### Isolation and serotyping of *S. enterica* subs. *enterica.*

Methodology for isolation of *Salmonella* spp. followed the International Organization for Standardization (ISO) standard 6579–1:2017 [17]. Samples were pre-enriched in BPW at 37°C for 24 hours. After pre-enrichment, 100 μl of aliquot was dropped to Modified Semisolid Rappaport-Vassiliadis (MSRV) (Difco) and incubated at 42°C for 24 hours. Positive colonies were then transferred onto Xylose Lysine Deoxycholate (XLD) Agar. The typical colonies of *Salmonella* spp. were pink with dark black centers and further biochemically confirmed by TSI, with *S. enterica* ATCC 14028 used as a positive control.

Genomic DNA was extracted using the whole cell boiled lysate technique [18]. *Salmonella* spp. was confirmed through PCR targeting the *invA* gene. Amplification was carried out using primers *invA*-F (GTGAAATTATCGCCACGTTCGGGCAA) and *invA*-R (TCATCGCACCGTCAAAGGAACC), which produced a 284 bp fragment as described by Kumar et al. [19]. PCR reactions were carried out with the DreamTaq PCR Master Mixes (Thermo Fisher Scientific, Waltham, MA, USA) in accordance with the manufacturer’s guidelines. The thermal cycling included an initial denaturation at 95°C for 2 minutes, followed by 35 cycles of denaturation at 95°C for 30 seconds, annealing at 58°C for 30 seconds, extension at 72°C for 60 seconds, and a final extension at 72°C was performed for 5 minutes. The PCR amplicons were separated by electrophoresis on a 1.5% agarose gel prepared in 1 × Tris-acetate-EDTA (TAE) buffer. The gel was stained with RedSafe™ nucleic acid staining solution (iNtRON Biotechnology, Seongnam, South Korea) and visualized under UV illumination using a gel documentation system (Omega Fluor, Aplegen, CA, USA) to verify the expected product size. *Salmonella* serotyping was performed using slide agglutination, based on the somatic and phase 1 and 2 flagellar antigen profiles, with commercial antisera (S&A reagent Lab Ltd., Bangkok, Thailand), in accordance with the Kauffmann–White Scheme [20].

#### Isolation and confirmation of *S. aureus.*

The method for *S. aureus* colony isolation followed the U.S. FDA BAM Chapter 12 protocol, with slight modifications [21]. In triplicate, a 100 μl aliquot of each serial dilution was spread onto Baird-Parker Agar (BPA) (Difco) using a sterile plate spreader. The plates were then inverted after the inoculum was absorbed and incubated at 37°C for 24–48 hours. Typical colonies of *S. aureus* on BPA appeared black, shiny, convex, and surrounded by a clear zone. Presumptive colonies were streaked onto CHROMagar^TM^ *Staph aureus* (CSA) (CHROMagar Microbiology, Paris, France) for selective differentiation [22]. Gram staining and biochemical confirmation using tube coagulase test with rabbit plasma (Bactident®, Merck Millipore, Germany) was performed according to the BAM protocol, with *S. aureus* ATCC 25923 used as a positive control.

*S. aureus* identification was confirmed through PCR using primers specific to the 23S rRNA gene (Staur4-F: ACGGAGTTACAAAGGACGAC and Staur6-R: AGCTCAGCCTTAA CGAGTAC), generating a 1250 bp product [23]. The PCR cycle includes an initial denaturation at 94°C for 5 minutes, followed by 30 cycles of denaturation at 94°C for 40 seconds, annealing at 64°C for 60 seconds, and extension at 72°C for 75 seconds, concluding with a final extension at 72°C for 7 minutes. The PCR amplicons were separated by electrophoresis on a 1.5% agarose gel prepared in 1 × TAE buffer. The gel was stained with RedSafe™ nucleic acid staining solution (iNtRON Biotechnology) and visualized under UV illumination using a gel documentation system (Omega Fluor) to verify the amplicon size.

#### Isolation and confirmation of *V. cholerae.*

Isolation of *V. cholerae* was performed using the U.S. FDA BAM Chapter 9 method [24]. Briefly, the suspension of homogenized sample described in the previous step was inoculated into 9 ml of Alkaline Peptone Water (APW) (Difco) and incubated at 37°C for 24 hours. If turbidity was observed, a loopful from positive tubes were then streaked onto Thiosulfate-Citrate-Bile Salts-Sucrose Agar (TCBS Agar) (Difco) and the plates then incubated at 37°C for 24 hours. Presumptive colonies of *V. cholerae* appeared as circular yellow colonies. The presumptive isolates were then streaked onto CHROMagar^TM^ *Vibrio* (CHROMagar Microbiology) and further confirmed using with TSI slant agar supplemented with 2% NaCl, with *V. cholerae* ATCC 14035 used as positive control.

Confirmation of *V. cholerae* was performed using PCR amplification targeting the outer membrane protein gene (*ompW*) with specific primers (*ompW*-F: CACCAAGAAGGTGA CTTTATTGTG and *ompW*-R: GAACTTATAACCACCCGCG), yielding an amplicon of 588 bp [25]. The PCR cycling includes an initial denaturation at 94°C for 5 minutes, followed by 30 cycles of denaturation at 94°C for 2 minutes, annealing at 50°C for 2 minutes, extension at 72°C for 30 seconds, and a final extension at 72°C for 7 minutes. The PCR amplicons were separated by electrophoresis on a 1.5% agarose gel prepared in 1 × TAE buffer. The gel was stained with RedSafe™ nucleic acid staining solution (iNtRON Biotechnology) and visualized under UV illumination using a gel documentation system (Omega Fluor) to verify the amplicon size.

#### Physical and chemical characteristics of dried shrimp.

The physical attributes of dried shrimp were characterized, including color, size, presence or absence of the shell, and shrimp species. These characteristics were then compared to the Thai Agricultural Standard (TAS 7012−2008) established by the National Bureau of Agricultural Commodity and Food Standards, Ministry of Agriculture and Cooperatives [13].

Chemical characteristics of baby dried shrimp were analyzed for the final products, consisting of stage-8 (n = 24) and dried shrimp from retail sources (n = 51). Approximately 200 grams per shrimp sample (n = 75) were submitted for analysis of salt content (NaCl, g/100 g), moisture content (g/100 g), and water activity (a_w_) by the Food Research and Testing Laboratory, Faculty of Science, Chulalongkorn University (https://cufoodtest.com/), which is accredited under ISO standards. The following standard methods were used: salt content was measured using an in-house method TC 043 based on AOAC (2023), 937.09; moisture content was determined using an in-house method based on AOAC (2023) 950.46(b); and water activity was assessed using an in-house method based on AOAC (2023) 978.18. In addition, pH value was determined as previously described [26]. Briefly, a 10 g sample of dried shrimp meat was homogenized in 90 ml of distilled water, the mixture filtered, and the pH measured using a portable water quality meter (Extech SDL100, Nashua, NH, USA) after 5 minutes of incubation at room temperature. The resulting data were then compared to the TAS 7012–2008 standards for salt content, moisture content, and water activity. Furthermore, pH levels were evaluated according to the Thai Industrial Standard (TIS 1003–2533) for dried shrimp [27].

### Statistical analysis

Negative binomial regression was used to test the association between levels of *E. coli* in dried shrimp (MPN/g) and the various factors and parameters such as stage of production, sample location, pH, color, size, presence of shell, NaCl content, percent moisture, water activity, total variable count, total and fecal coliforms, *S. aureus*, *Salmonella* spp., and *V. cholerae*. The negative binomial regression model was applied to the *E. coli* MPN/g data because this distribution can accommodate the common problem of overdispersion (variance greater than the mean) associated with bacterial counts per mass or volume. To account for potentially correlated microbial data when repeatedly sampling from the same locations, we adjusted the *p*-values and confidence intervals of our regression analyses using a robust variance estimator. Initially, we screened all independent variables for potential significance using univariable regression models. Employing the *p*-value as the criterion for initial inclusion, we then used a backward stepwise algorithm to construct multivariable fixed-effects negative binomial and logistic regression models for the dried shrimp analyses. Variables were retained in the final models if their likelihood ratio test was ≤ 0.05. All statistical analyses were conducted using Stata Version 18.0 (StataCorp, College Station, TX, USA).

### Ethical approval, IAUCC and IBC

Ethical approval for the survey questionnaire was granted (No. 187/67) from 20/8/2024-19/8/2025 by Chulalongkorn University, Bangkok, Thailand. The study design was also reviewed and approved by the Institutional Animal Care and Use Committee at the Faculty of Veterinary Science, Chulalongkorn University, Bangkok, Thailand (Approval ID No. 2431024). Additionally, the laboratory use of pathogens and chemicals in the study was approved by the Faculty of Veterinary Science Institutional Biosafety Committee (CU-VET-IBC 2431013).

## Results

### Demographic characteristics of dried shrimp production

In this study, two-thirds of the participating households (farmers) involved in dried shrimp production were from Surat Thani, while one-third were from Nakhon Si Thammarat (Table 1). Farmers had varying levels of experience in dried shrimp production, ranging from 4 to more than 10 years. Many of these farmers (8/12, 66.7%) possessed over five years of experience in this industry. The primary species of shrimp produced for drying were *Parapenaeus longipes* (Kung Sai) and *Acetes* spp. (Krill), whereby both species represented 33.3% of the total production from the sampled households. Over 60% of households used nets for shrimp harvesting. Sorting shrimp after harvesting (Stage 2) was not mandatory, as some farmers preferred to sort them during the grading stage (Stage 7). The majority of households (66.7%) wash the shrimp one to two times, while the remaining 33.3% wash it more than five times. All farmers (100%) use boiling for sterilization, which lasts between 30 and 60 minutes, with the range of water temperatures reported as 95°C to 100°C. Salt was added at a concentration of approximately 500 g salt/10 kg shrimp during processing by all farmers. Only one-third of the farmers used coloring agents in shrimp processing. Shrimp drying methods varied, including drying on the bare floor, aluminum sheets, or wooden boards. Farmers reported that they did not directly measure the ambient temperature during drying via solar radiation, but it was known that surface temperatures varied from 35°C to 50°C depending on the type of drying material being used. Nearly 70% of farmers required two days to dry the shrimp. The option of removing the shell depended on the customer’s demand. The preferred season for dried shrimp production was summer (mid-February to mid-May), with 100% of farmers operating during this time. While dried shrimp production occurs year-round, summer is the preferred season for all farmers (100%), with an average production frequency of 10–20 times per year. Different storage methods were used to preserve baby dried shrimp, including refrigeration at 2–8°C, airtight plastic bags, and closed-lid glass jars. Most farmers (66.7%) store the shrimp for one week before offering to sell to consumers, while 33.3% keep the product for up to three weeks prior to selling. Regarding market dynamics, 75% of farmers sell their products directly to consumers, while the remainder rely on middlemen. Notably, 66.7% of farmers report a production output exceeding 10 kg per cycle, with estimated costs ranging from 400 to 500 Thai Baht per kg.

**Table 1:** Descriptive statistics of stages of dried shrimp production across 12 households in two different Provinces of Thailand.

Stage of production	Category	N (%)	Stage of production	Category	N (%)
Demographic information	Location		Processing (Stage 4 – cooking)	Coloring added to the shrimp	
	Nakhon Si Thammarat Surat Thani	4 (33.3) 8 (66.7)		No Yes	8 (66.7) 4 (33.3)
	Years of experience in production		Processing (Stage 5 – drying)	Methods for drying shrimp	
	< 5 ≥ 5-10 > 10	4 (33.3) 4 (33.3) 4 (33.3)		Drying on the floor without cover Drying on aluminum sheets Drying on wooden boards	4 (33.3) 4 (33.3) 4 (33.3)
Harvest	Types of shrimp species used for drying			Duration (in days) required to dry the shrimp	
	*Parapenaeus longipes* (Kung Sai) *Acetes* spp. (Krill) *Litopenaeus vannamei* (White leg shrimp) *Penaeus merguiensis* (Red tail shrimp)	4 (33.3) 4 (33.3) 2 (16.7) 2 (16.7)		1 2 >2	4 (33.3) 8 (66.7) 0 (0)
	Technique used for harvesting shrimp		Processing (Stage 6 – peeling)	Process of removing the shrimp shell	
	Net Swing	8 (66.7) 4 (33.3)		No Yes	8 (66.7) 4 (33.3)
Processing (Stage 2 – sorting and 7 – grading)	Method of sorting dried shrimp by size			Method of removing shrimp shells	
	None After harvesting After drying	4 (33.3) 4 (33.3) 4 (33.3)		No removal Manual removal Machine/automatic removal	4 (33.3) 4 (33.3) 4 (33.3)
	Techniques for sorting shrimp by size (n =8)		Final products	Optimal season for dried shrimp production	
	Manual Automatic machine	8 (100) 0 (0)		Summer Winter Rainy	12 (100) 0 (0) 0 (0)
Processing (Stage 3 – washing)	Washing the shrimp before sterilizing			Duration (weeks) of dried shrimp storage before selling	
	No Yes	0 (0) 12 (100)		1-2 3-4	8 (66.7) 4 (33.3)
Processing (Stage 4 – cooking)	Number of washes before sterilization			Number of dried shrimp production cycles per year	
	1-2 3-5 >5	8 (66.7) 0 (0) 4 (33.3)		<10 ≥ 10	0 (0) 12 (100)
	Techniques for cooking or sterilizing shrimp			The amount (kg) of dried shrimp that produce/time	
	Boiling Steam	12 (100) 0 (0)		<10 ≥ 10-20 >20	4 (33.3) 4 (33.3) 4 (33.3)
	Duration of shrimp boiling (minutes)			Method of storing dried shrimp before selling	
	1-30 > 30-50 > 50	4 (33.3) 4 (33.3) 4 (33.3)		Refrigerator (2-8°C) Airtight plastic bag Closed lid glass jar	4 (33.3) 4 (33.3) 4 (33.3)
	The addition of salt (NaCl)/10 kg			Process for selling dried shrimp	
	No Yes	0 (0) 12 (100)		Selling by themselves Selling through a middleman	9 (75.0) 3 (25.0)
	Added amount of salt (grams)/10 kg			Estimated cost (Thai Baht)/kg of dried shrimp	
	< 500 ≥ 500	8 (66.7) 4 (33.3)		300-400 400-500	4 (33.3) 8 (66.7)

Note: Households were chosen based on their ongoing production and willingness to participate. These 12 households account for 20% of all producers in the study area.

### Physical and chemical characteristics of baby dried shrimp

A variety of shrimp species was collected at different stages of production, resulting in species differences between production-stage samples and final dried products. The composition of species of shrimp during production included *Parapenaeus longipes* (n = 8), *Macrobrachium lanchesteri* (n = 8), *Litopenaeus vannamei* (n = 6), and *Penaeus longistylus* (n = 2). Similarly, the species composition of the final dried products at retail included *P. merguiensis* (n = 7), *M. lanchesteri* (n = 6), *P. longipes* (n = 6), *L. vannamei* (n = 6), *Acetes* spp. (n = 1), *Pleoticus muelleri* (n = 1), and unidentified species (n = 24). The shrimp sizes ranged from small (n = 26, 34.7%), medium (n = 46, 61.3%), to large (n = 3, 4.0%), and the colors varied, including natural (n = 12, 16.0%), orange colored (n = 49, 63.3%), and red colored (n = 14, 18.7%) (Table 2). Most of the samples retained their shell (n = 46, 61.3%).

**Table 2:** Physical and chemical characteristics of dried shrimp samples (n = 75).

Category	Location	Species	Physical characteristic (n)			Chemical characteristic (Average ± S. D.)			
			Size	Color	Present of the shell	Salt content (g/100 g)	Moisture (g/100 g)	Water activity	pH
Production	Tha Sala District, Nakhon Si Thammarat (n = 8)	*Parapenaeus longipes* (8)	Medium (8)	Orange (2) Red (6)	0	6.3 (4.1)	27.6 (9.6)	0.76 (0.08)	8.19 (0.27)
	Kanchanadit District, Surat Thani (n = 8)	*Macrobrachium lanchesteri* (8)	Small (8)	Orange (8)	6	9.9 (3.2)	20.5 (4.9)	0.65 (0.04)	7.75 (0.11)
	Mueang District, Surat Thani (n = 8)	*Litopenaeus vannamei* (6) *Penaeus longistylu*s (2)	Medium (8)	Natural (4) Orange (4)	2	3.0 (0.7)	25.3 (13.5)	0.76 (0.12)	8.14 (0.58)
	Total (n = 24)		Small (8) Medium (16)	Natural (4) Orange (14) Red (2)	8	6.4 (4.1)	24.5 (10.0)	0.72 (0.08)	8.03 (0.41)
Final product	1^st^ store, Samut Sakhon (n = 6)	*Acetes* spp. (1); *Macrobrachium lanchesteri* (2); *Penaeus merguiensis* (3)	Small (3) Medium (3)	Natural (1) Orange (3) Red (2)	4	5.5 (3.1)	27 (8.5)	0.73 (0.10)	7.52 (0.40)
	2^nd^store, Samut Sakhon (n = 4)	*M. lanchesteri* (2); *P. merguiensis* (2)	Small (2) Medium (2)	Natural (2) Red (2)	4	9.4 (6.3)	29.2 (10.8)	0.72 (0.06)	7.69 (0.27)
	3^rd^ store, Samut Sakhon (n = 6)	*M. lanchesteri* (2); *L. vannamei* (1); *P. merguiensis* (2); *Pleoticus muelleri* (1)	Small (2) Medium (2) Large (2)	Natural (2) Orange (4)	3	7.1 (7.1)	36.5 (11.3)	0.82 (0.09)	8.0 (0.20)
	Nonthaburi (n = 2)	NA (2)	Small (1) Large (1)	Orange (1) Red (1)	2	6.0 (4.4)	33.2 (19)	0.74 (0.15)	8.05 (0.21)
	Trang (n = 2)	NA (2)	Medium (1) Large (1)	Natural (1) Orange (1)	1	8.0 (5.5)	27.2 (2.3)	0.74 (0.04)	7.85 (0.07)
	Lat Krabang District, Bangkok n = 7)	*P. longipes* (1); *L. vannamei* (5); NA (1)	Small (1) Medium (6)	Orange (7)	7	9.5 (2.7)	33.6 (9.0)	0.74 (0.05)	8.09 (0.25)
	Lat Krabang District, Bangkok (n = 4)	*P. longipes* (2); NA (2)	Small (2) Medium (2)	Orange (4)	3	15.3 (9.4)	36.0 (3.9)	0.78 (0.06)	7.85 (0.16)
	Thawi Watthana District, Bangkok (n = 10)	*P. longipes* (2); NA (8)	Small (2) Medium 8)	Natural (1) Orange (7) Red (2)	8	7.3 (5.2)	39.8 (10.2)	0.82 (0.07)	7.84 (0.18)
	Bang Khun Si, Bangkok Noi District, Bangkok (n = 8)	*P. longipes* (1); NA (7)	Small (5) Medium (3)	Orange (7) Red (1)	6	9.7 (6.3)	37.8 (11.5)	0.78 (0.08)	7.83 (0.13)
	Don Chedi District, Suphan Buri (n = 2)	NA (2)	Medium (2)	Natural (1) Orange (1)	0	4.1 (1.5)	29.1 (1.3)	0.81 (0)	7.89 (0.06)
	Total (n = 51)		Small (18) Medium (30) Large (3)	Natural (8) Orange (35) Red (8)	38	8.4 (5.7)	34.4 (10.1)	0.77 (0.08)	7.84 (0.27)
Grand total (n = 75)			Small (26) Medium (46) Large (3)	Natural (12) Orange (49) Red (14)	46	7.8 (5.3)	31.2 (11.1)	0.76 (0.09)	7.90 (0.33)

Note: The size of baby dried shrimp was categorized as small (more than 501 per 100 g), medium (251–500 per 100 g), and large (51–250 per 100 g); S. D.: standard deviation; NA: the species of shrimp cannot be identified.

The average and standard deviation of salt content (7.8 ± 5.3 g/100g), moisture content (31.2 ± 11.1 g/100g), water activity (0.8 ± 0.09), and pH (7.9 ± 0.33) were recorded. Due to the collection of different shrimp species from both production and retail sites, their physicochemical characteristics were reported separately rather than being compared directly. There were higher average values and wider ranges of salt and moisture content for samples from retail sites (8.4 ± 5.7 g/100g and 34.4 ± 10.1 g/100g, respectively) compared to samples from production sites (6.4 ± 4.1 g/100g and 24.4 ± 10.0 g/100g, respectively). The pH levels varied between sites, with samples from production sites exhibiting an average value of 8.0 ± 0.41 and samples from retail sites exhibiting average values of 7.8 ± 0.27. According to TIS standards, 50% (12 out of 24) of the samples from the processing stage and 19.1% (9 out of 47) of the final dried product samples exceeded the acceptable pH levels (Table 3). Despite the variation in pH, the water activity remained consistent, ranging from 0.7 to 0.8 for all samples.

**Table 3:** Compliance of dried shrimp physicochemical parameters with TAS at production sites and retail markets.

Parameter	Standard	Compliant (%)		
		Production site	Retail	Overall
Salt (%)	≤ 7	15 (62.5)	29 (56.9)	44 (58.7)
Moisture (%)	≤ 30	19 (79.2)	17 (33.3)	36 (48.0)
Water activity	≤ 0.75	16 (66.7)	22 (43.1)	38 (50.7)
pH*	≤ 8.0	12 (50.0)	38 (80.9)	50 (70.4)

Note: Compliance criteria followed the TAS 7012–2008; Due to missing measurements, pH compliance was calculated from the available samples (n = 20 for production sites; n = 71 overall).

### Environmental parameters

Environmental parameters varied across the production sites (Table 4). The overall averages (± S.D.) for maximum wind speed, average wind speed, relative humidity, and ambient air temperature were 7.0 ± 5.5 m/s, 2.0 ± 0.7 m/s, 73.6 ± 8.2%, and 33.7 ± 2.5°C, respectively. Tha Sala, Nakhon Si Thammarat recorded the highest maximum wind speed at 9.7 ± 8.4 m/s, compared to Surat Thani province. Kanchanadit district in Surat Thani recorded the highest average wind speed at 2.6 ± 0.7 m/s. Relative humidity was highest in Mueang district, Surat Thani, at 78.7 ± 5.9%, while Kanchanadit district, Surat Thani, showed greatest variability (72.3 ± 10.6%) compared to the other sites. The ambient air temperature was the highest in Tha Sala district, Nakhon Si Thammarat (34.8 ± 3.2°C). In addition, the Thai Meteorological Department (TMD) recorded data for dry temperature at 25.9 ± 1.2°C, maximum temperature at 34.5 ± 1.9°C, minimum temperature at 25.1 ± 1.0°C, and 24-hour cumulative rainfall as 0.4 ± 0.9 mm.

**Table 4:** Environmental parameters collected from the production sites of dried shrimp (n = 24).

Location	Average (± S.D.)							
	Maximum wind speed (m/s)	Wind speed (m/s)	Relative humidity (%)	Ambient air temp (°C)	Dry temp* (°C)	Maximum temp* (°C)	Minimum temp* (°C)	Rain* (mm/day)
Tha Sala district, Nakhon Si Thammarat province (n = 8)	9.7 **±** 8.4	2.0 **±** 0.5	69.7 **±** 5.1	34.8 **±** 3.2	25.4 **±** 1.3	34.5 **±** 2.1	25.0 **±** 1.3	0.0 **±** 0.0
Kanchanadit district, Surat Thani province (n = 8)	8.0 **±** 2.5	2.6 **±** 0.7	72.3 **±** 10.6	33.9 **±** 2.3	26.7 **±** 1.1	35.5 **±** 1.5	25.4 **±** 0.9	0.2 **±** 0.3
Mueang district, Surat Thani province (n = 8)	3.5 **±** 0.9	1.5 **±** 0.3	78.7 **±** 5.9	32.5 **±** 1.6	25.7 **±** 0.9	33.4 **±** 1.6	24.8 **±** 0.7	1.0 **±** 1.3
**Grand total**	7.0 **±** 5.5	2.0 **±** 0.7	73.6 **±** 8.2	33.7 **±** 2.5	25.9 **±** 1.2	34.5 **±** 1.9	25.1 **±** 1.0	0.4 **±** 0.9

Note: A total of 24 environmental measurements were collected from 12 households (duplicate measurements per household) and grouped into three districts; S.D.: standard deviation; temp: temperature; * these environmental data were collected from the Thai Meteorological Department.

Most of the on-site environmental data showed no significant correlations with microbial loads (p > 0.05) (Table 5). Among the environmental variables recorded by the TMD, ambient dry temperature exhibited a significant negative correlation with fecal coliforms (*ρ* = –0.608, p = 0.036), which indicates that higher dry temperatures corresponded with lower fecal coliform levels during dried shrimp production. Additionally, relative humidity demonstrated a significant positive correlation with *E. coli* (*ρ* = 0.671, p = 0.017), suggesting that dried shrimp production conducted under more humid conditions tended to exhibit higher *E. coli* loads.

**Table 5:** Spearman’s correlation (ρ) and associated p-values (p) between environmental parameters and mean log_10_ microbial loads per fishing round.

Environmental parameter	TVC, ρ(p)	Total coliform, ρ(p)	Fecal coliform, ρ(p)	*E. coli*, ρ(p)	*S. aureus*, ρ(p)
On-site collection					
Maximum wind speed (m/s)	–0.044 (0.892)	0.432 (0.161)	–0.232 (0.469)	–0.319 (0.312)	0.102 (0.751)
Wind speed (m/s)	–0.044 (0.893)	–0.393 (0.206)	–0.252 (0.430)	–0.531 (0.075)	–0.043 (0.894)
Relative humidity (%)	0.393 (0.206)	0.266 (0.404)	0.351 (0.263)	0.671 (0.017*)	–0.462 (0.130)
Ambient air temp (˚C)	–0.480 (0.114)	0.252 (0.430)	–0.608 (0.036*)	–0.357 (0.255)	0.382 (0.221)
Thai meteorological data					
Dry temp (˚C)	–0.399 (0.199)	–0.158 (0.623)	0.285 (0.370)	0.221 (0.489)	0.027 (0.934)
Maximum temp (˚C)	–0.070 (0.829)	–0.018 (0.957)	0.130 (0.687)	0.154 (0.633)	0.054 (0.868)
Minimum temp (˚C)	0.280 (0.379)	0.074 (0.820)	0.049 (0.879)	0.098 (0.762)	–0.027 (0.934)
Rain (mm/day)	–0.429 (0.165)	0.125 (0.699)	0.458 (0.135)	0.208 (0.516)	–0.307 (0.332)

Note: * indicate statistical significance at *p* < 0.05.

### Presence of total viable count, total coliforms, fecal coliforms, and *E. coli*

The overall prevalence of detectable total viable counts, total coliforms, fecal coliforms, and *E. coli* in this study was 97.0% (195/201), 94.0% (189/201), 41.3% (83/201), and 26.4% (53/201), respectively (Tables 6, 7). The average concentrations (median) of total viable count, total coliforms, fecal coliforms, and *E. coli* were 1.09 × 10^10^ (6.60 × 10^4^), 7,638.6 (1.20 × 10^4^), 895.7 (3.0), and 394.8 (3.0) MPN/g. Furthermore, the prevalence of samples that exceeded standard limits for these bacterial indicators was 47.3% for total viable count, 79.1% for total coliforms, 21.9% for fecal coliforms, and 26.4% for *E. coli*. Total coliforms were consistently detected across almost all processing stages, including fishing, sorting, washing, drying, peeling, grading, and packaging stages, with a prevalence of 100%. Only the cooking stage showed a slight reduction in prevalence of total coliforms (95.8%), along with 78.4% for retail market dried shrimp.

**Table 6:** The distribution of total viable count among dried shrimp and water samples (n = 201).

Category	Stage	Total viable count		
		Positive (%)	Mean concentration (±median) log10 CFU/g	Exceeding Standard (%)
Pre-harvest	Water sample (n = 24)	24 (100)	5.75 × 10³ (1.65 × 10³)	0 (0)
	Stage 1-fishing (n = 24)	24 (100)	8.82 × 10¹⁰ (3.34 × 10⁶)	20 (83.3)
Processing stage	Stage 2-sorting (n = 8)	8 (100)	4.23 × 10⁶ (6.65 × 10⁵)	7 (87.5)
	Stage 3-washing (n = 18)	18 (100)	2.00 × 10⁸ (6.38 × 10⁶)	14 (77.8)
	Stage 4-cooking (n = 24)	18 (75.0)	2.97 × 10³ (1.03 × 10³)	0 (0)
	Stage 5-drying (n = 16)	16 (100)	3.87 × 10⁸ (1.24 × 10⁴)	6 (37.5)
	Stage 6-peeling (n = 6)	6 (100)	2.27 × 10⁶ (5.79 × 10⁴)	3 (50.0)
	Stage 7-grading (n = 6)	6 (100)	1.30 × 10⁹ (1.05 × 10⁴)	2 (33.3)
Final products	Stage 8-packaging and labelling (n = 24)	24 (100)	9.85 × 10⁸ (2.03 × 10⁴)	10 (41.7)
	Stage 9-market dried shrimp (n = 51)	51 (100)	5.66 × 10⁸ (4.26 × 10⁶)	33 (64.7)
**Grand total (n = 201)**		195 (97.0)	1.09 × 10¹⁰ (6.60 × 10⁴)	95 (47.3)

Note: The concentration of total viable count should not exceed 5.00 log10 CFU/g (10^5^ CFU/g).

**Table 7:** The distribution of total coliforms, fecal coliforms, and *E. coli* among dried shrimp and water samples (n = 201).

Category	Stage	Total coliforms			Fecal coliforms			*E. coli*		
		Positive (%)	Mean concentration (±median)	Exceeding Standard (%) ^a^	Positive (%)	Mean concentration (±median)	Exceeding Standard (%) ^b^	Positive (%)	Mean concentration (±median)	Exceeding Standard (%) ^c^
Pre-harvest	Water sample (n = 24)	24 (100)	5,094.6 (3,050)	15 (62.5)	20 (83.3)	780.8 (180.0)	13 (54.2)	17 (70.8)	36.9 (8.3)	17 (70.8)
	Stage 1-fishing (n = 24)	24 (100)	12,000.0 (12,000.0)	24 (100)	22 (91.7)	1,176 (121.5)	12 (50.0)	17 (70.8)	255.3 (9.2)	17 (70.8)
Processing stage	Stage 2-sorting (n = 8)	8 (100)	11,875.0 (12,000.0)	8 (100)	7 (87.5)	148.0 (23.0)	2 (25.0)	1 (12.5)	3.1 (3.0)	1 (12.5)
	Stage 3-washing (n = 18)	18 (100)	12,000.0 (12,000.0)	18 (100)	14 (77.8)	984.7 (9.2)	5 (27.8)	5 (27.8)	10.6 (3.0)	5 (27.8)
	Stage 4-cooking (n = 24)	23 (95.8)	1,821.6 (430.0)	8 (33.3)	10 (41.7)	5.2 (3.0)	0 (0)	4 (16.7)	3.1 (3.0)	4 (16.7)
	Stage 5-drying (n = 16)	16 (100)	8,480.6 (12,000.0)	13 (81.3)	3 (18.8)	903.8 (3.0)	2 (13.0)	2 (12.5)	902.6 (3.0)	2 (12.5)
	Stage 6-peeling (n = 6)	6 (100)	10,600.0 (12,000.0)	6 (100.0)	3 (50.0)	4,352 (1,052)	3 (50.0)	3 (50.0)	4,251.5 (751.5)	3 (50.0)
	Stage 7-grading (n = 6)	6 (100)	4,837.3 (2,420.0)	3 (50.0)	0 (0)	3.0 (3.0)	0 (0)	0 (0)	3.0 (3.0)	0 (0)
Final products	Stage 8-packaging and labelling (n = 24)	24 (100)	7,168,4 (11,500.0)	19 (79.2)	4 (16.7)	1,007 (3.0)	2 (8.3)	4 (16.7)	1,006.5 (3.0)	4 (16.7)
	Stage 9-market dried shrimp (n = 51)	40 (78.4)	5,557.1 (2,400.0)	30 (58.8)	0 (0)	3.0 (3.0)	0 (0)	0 (0)	3.0 (3.0)	0 (0)
**Grand total (n = 201)**		189 (94.0)	7,207.7 (12,000.0)	144 (71.6)	83 (41.3)	651.2 (3.0)	39 (19.4)	53 (26.4)	356.1 (3.0)	53 (26.4)

Note: The microbiological standard limits used to determine “exceeding standard” were: (a) total coliforms >1,000 MPN/g, (b) fecal coliforms >100 MPN/g, and (c) *E. coli* >3 MPN/g.

The prevalence of fecal coliforms and *E. coli* exhibited a decreasing trend across the processing stages. The highest *E. coli* prevalence was observed for the fishing stage and in water samples (70.8% positive), followed by peeling (50.0%), washing (27.8%), cooking (16.7%), packaging and labelling (16.7%), cleaning (12.5%), and drying (12.5%); no *E. coli* was detected during the grading stage and for retail market dried shrimp. Of the 53 positive samples, two isolates were collected per sample, resulting in a total of 106 isolates for further analysis. When examined seasonally, *E. coli* was not detected in any samples collected during the winter season. In contrast, 17 positive samples were identified in the summer season and 36 positive samples in the rainy season.

### Prevalence of *S. enterica* subs. *enterica*, *S. aureus*, and *V. cholerae*

Out of the 201 samples, *S. enterica* subs. *enterica*, *S. aureus*, and *V. cholerae* were detected at 1.0% (2/201), 2.5% (5/201), and 2.0% (4/201) of the samples, respectively. No contamination of *Salmonella* spp., *S. aureus*, or *V. cholerae* was found in samples collected during the winter season. In the summer, only one sample tested positive for *Salmonella* spp. However, a wider range of bacterial contamination in shrimp samples was observed during the rainy season, including *Salmonella* spp. (n = 1), *S. aureus* (n = 5), and *V. cholerae* (n = 3). *S. enterica* subs. *enterica* was detected in the pre-harvest stage, with positive samples of water (n = 1) and shrimp from stage 1-fishing (n = 1). *S. aureus* was present in shrimp for only several stages, including fishing (n = 1), washing (n = 2), packaging (n = 1), and retail market (n = 1). In contrast, *V. cholerae* was detected in the water (n = 2), stage 1-fishing (n = 1), and during the processing stage 4-cooking (n = 1). Serotyping of *S. enterica* subs. *enterica* identified two distinct serovars: Tallahassee and Mount Pleasant. The average plate count for positive *S. aureus* samples was 1.5 x 10⁴ CFU/g, with all these positive samples exceeding the microbiological limit of 100 CFU/g set by TAS. Although *V. cholerae* was detected in our samples, the isolates were not serotyped; therefore, their pathogenic potential (e.g., O1/O139 serogroups) is unknown, representing a limitation of this study.

Negative binomial regression analysis identified variables associated with the presence of *E. coli* in dried shrimp (Table 8). These univariate regression results may be confounded and should be interpreted with caution, emphasizing that the multivariable model likely provides a more valid assessment of these risk factor associations. Multivariable regression revealed that pH, sampling location, coloring shrimp, and fecal coliforms were either associated with *E. coli* concentrations or helped control for potential confounding (Table 9). For example, although pH showed a negative association with *E. coli* in the univariate analysis, this relationship reversed to a positive association for the main effect after adjusting for the other variables in the final multivariable model (i.e., Province where samples were taken, coloring shrimp, and a pH × color interaction with their main effects). Hence, although the results in Table 9 indicate there were no significant differences between the various Provinces and the occurrence of *E. coli* relative to their referent (Nakhon Si Thammarat), inclusion of this variable and the other variables in the final model allowed us to control for strong confounding regarding pH. In addition, the direction and magnitude of the association between *E. coli* concentration and pH significantly differed between colored and non-colored shrimp given the significant interaction terms, indicating strong effect measure modification between pH and adding color regarding *E. coli* levels. Specifically, for non-colored shrimp, the model indicates that there was a 16.2 times (Θ^2.783^ = 16.2) greater concentration of *E. coli* in shrimp meat with a pH > 8 compared shrimp meat at a pH < 8, but this significant effect of pH was not present once the shrimp was colored either red (Θ^2.783 + 0.004-2.730^ = 1.14) or orange (Θ^2.783 + 0.004-2.731^ = 1.14). These results suggest that the effect of pH on *E. coli* was significantly influenced by either the chemistry of these two food coloring dyes, the process used to color the shrimp, or other some other related process or chemical reaction.

**Table 8:** Univariable negative binomial regression of *E. coli* and predictors.

Predictors	Coefficient	Std. Err.	C.I.	*p*-value
Production				
Pre-harvest	Ref.			
Processing	1.262	0.428	0.434-2.100	0.003
Final products	0.797	0.431	−0.048-1.642	0.064
pH*	−4.685	0.392	−5.452-(−3.917)	<0.0001
pH standard*				
< 8	Ref.			
> 8	−4.190	0.586	−5.338-(−3.041)	<0.0001
Location				
Nakhon Si Thammarat	Ref.			
Samut Sakhon	−3.894	0.334	−4.545-(−3.329)	<0.0001
Other provinces^#^	−5.443	0.335	−6.100-(−4.787)	<0.0001
Color				
Natural (no color)	Ref.			
Red	−5.751	0.400	−6.535-(−4.968)	<0.0001
Orange	−5.753	0.282	−6.305-(−5.202)	<0.0001
Size				
Small	Ref.			
Medium	4.295	0.329	3.650-4.941	<0.0001
Large	−1.091	1.113	−3.273-1.091	0.327
Shell				
Absent	Ref.			
Present	4.445	0.441	3.581-5.309	<0.0001
NaCl content*	−0.333	0.400	−0.402-(−0.266)	<0.0001
NaCl standard (%)*				
Met (<0.7)	Ref.			
Did not meet (>0.7)	−5.212	0.484	−6.162-(−4.262)	<0.0001
Moisture*	−0.189	0.017	−.2222-(−0.157)	<0.0001
Moisture standard (%)*				
Met (<30)	Ref.			
Did not meet (>30)	−5.412	0.443	−6.280-(−4.544)	<0.0001
Water activity*	−5.880	26.691	−58.193-46.432	0.826
Water activity standard*				
Met (<0.75)	Ref.			
Did not meet (>0.75)	4.796	0.463	3.888-5.703	<0.0001
Total viable count (CFU/g)	−2.19 × 10^-12^	1.13 × 10^-12^	−4.40 × 10^-12^ - 1.43 × 10^-14^	0.052
Total viable count standard (CFU/g)				
Met (<10^5^)	Ref.			
Did not meet (>10^5^)	4.129	0.278	3.585-4.674	<0.0001
Total coliforms (MPN/g)	0.0004	0.00003	0.0003-0.0004	<0.0001
Total coliforms standard (MPN/g)				
Met (<10^3^)	Ref.			
Did not meet (>10^3^)	3.840	0.340	3.174-4.507	<0.0001
Fecal coliforms (MPN/g)	0.001	0.0001	0.0009 −0.0013	<0.0001
Fecal coliforms standard (MPN/g)				
Met (<100)	Ref.			
Did not meet (>100)	5.923	0.229	5.474-6.371	<0.0001
*S. aureus* (CFU/g)	−1.60 × 10^−6^	8.05 × 10^−7^	−3.18 × 10^−6^- (−1.93 × 10^−8^)	0.047
*S. aureus*				
Absent	Ref.			
Present	−4.802	1.088	−6.934-(−2.669)	<0.0001
*Salmonella*				
Absent	Ref.			
Present	−3.443	1.684	−6.743-(−0.142)	0.041
*V. cholerae*				
Absent	Ref.			
Present	2.159	1.054	0.0931-4.224	0.041

Ref: reference group; Std Err.: standard error; C.I.: confidence interval; Ref: reference; Other provinces comprised of Nonthaburi, Trang, Bangkok, Surat Thani, and Suphan Buri; Note: only observed on final products.

**Table 9:** Multivariable negative binomial regression of *E. coli* and predictors.

Predictors	Coefficient	Std. Err.	C.I.	*p*-value
pH standard*				
< 8	Ref.			
> 8	2.783	0.404	1.940-3.522	<0.0001
Location				
Nakhon Si Thammarat	Ref.			
Samut Sakhon	0.002	0.502	−0.981-(0.986)	0.996
Other provinces^#^	0.003	0.221	−0.429-(0.435)	0.989
Color				
Natural (no color)	Ref.			
Red	0.004	0.337	−0.656-(0.664)	0.991
Orange	0.004	0.278	−0.541-(0.550)	0.987
pH*Color				
pH > 8*Red	−2.730	0.700	−4.102-(−1.359)	<0.0001
pH > 8*Orange	−2.731	0.452	−3.617-(−1.845)	<0.0001
Fecal coliforms (MPN/g)	0.0006	0.00003	0.0006-0.0007	<0.0001
Intercept	1.090	0.330	0.442-1.773	0.001

AIC = 311.6178.

Ref: reference group; Std Err.: standard error; C.I.: confidence interval; Ref: reference; Other provinces comprised of Nonthaburi, Trang, Bangkok, Surat Thani, and Suphan Buri; AIC: Akaike Information Criterion.

## Discussion

**Fig 2:**
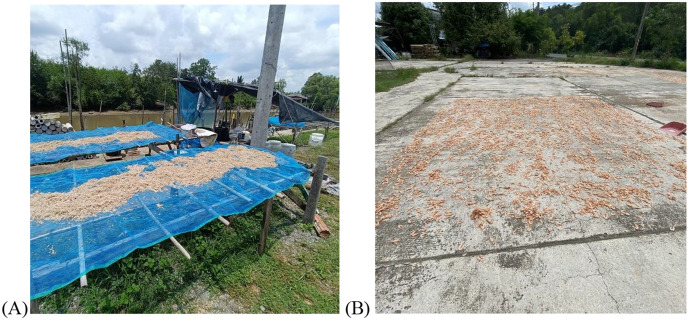
Method of drying shrimp using traditional artisanal techniques: **(A) Shrimp being sun-dried on a blue net placed over a bamboo table, and** **(B) Shrimp being sun-dried directly on a concrete floor surface.**

Traditional household farming (smaller scale, low resource) is the primary method for dried shrimp production in Thailand, with a large majority (around 70%) of farmers possessing at least five years of experience. The main baby shrimp species utilized are *Parapenaeus longipes* (Kung Sai) and *Acetes* spp. (krill). Kung Sai is a widely preferred dried shrimp in Thai cooking, used in dishes like omelets with sweet fish sauce, rolled noodles, Pad Thai, and spicy salads, as well as shrimp patties. Krill is commonly found in noodles and papaya salads. Medium- and large-sized dried shrimp are often processed with the shell removed, while small-sized shrimp are generally processed without removing the shell. Considering the frequent use of these dried shrimps in Thai cuisine, including raw consumption, evaluating their quality and food safety would help promote public health. Despite the popularity of these dried commodities in Thai cuisine, there exists a lack of established national guidelines and practices in dried shrimp production that maximize food safety and quality. However, we observed a significant decontamination step employed by nearly 70% of farmers which involved boiling for sterilization, followed by a two-day drying period without controlled temperature or humidity conditions (Fig 2). According to FAO and ILO, drying should occur in a clean and protected environment, devoid of filth flies, other vertebrate pests, and excessive fugitive dust [6]. The stated reasons for drying included facilitating shell removal and controlling bacterial growth, and the primary season when dried shrimp processing occurred was during the hot summer months in these regions of Thailand (supplementary Table 1).

The physical and chemical characteristics of the shrimp were characterized in accordance with TAS [13]. However, pH values were evaluated based on TIS 1003–2533 for dried shrimp [27]. One third of the farmers used coloring dyes in their shrimp processing, and our evaluation of the physical appearance of the dried baby shrimp revealed that the majority of samples were colored orange (63.3%) and red (18.7%) (Table 1). The color variation visually observed in the dried shrimp samples was primarily due to the addition of coloring dyes. The main pigment of shrimp, astaxanthin, undergoes significant degradation during the drying process and storage [28]. This degradation might lead to some sellers’ practice of adding colorants to enhance the appearance of their products. Additionally, the TAS food safety/quality limit for salt content (≤ 7% by weight), moisture content (≤ 30% by weight) and water activity (≤ 0.75) was only met for 41.3%, 52.0%, 49.3% of the samples, respectively. These moderate compliance levels indicate that a substantial proportion of retail products do not comply with established TAS standards, likely reflecting inconsistencies in processing methods and quality control metrics.

This study also quantified total coliforms, fecal coliforms, and *E. coli* in shrimp at the various stages involved in producing dried shrimp for retail distribution. Hygiene criteria for dried shrimp, such as total viable count, yeast and mold counts, *E. coli* levels, and the presence of *Salmonella* spp., *Clostridium perfringens*, and *S. aureus*, have been recommended by TAS for testing the quality of dried shrimp [13]. Although *C. perfringens* is included in the recommended tests for dried shrimp quality, it was not assessed in this study due to methodological limitations and the focus on aerobic and common foodborne bacteria. Similarly, yeasts and molds were not evaluated in this study, as their detection necessitates distinct analytical methods. Including these analyses in future research would offer a more thorough evaluation of the microbiological quality and safety of sun-dried shrimp products.

**Fig 3:**
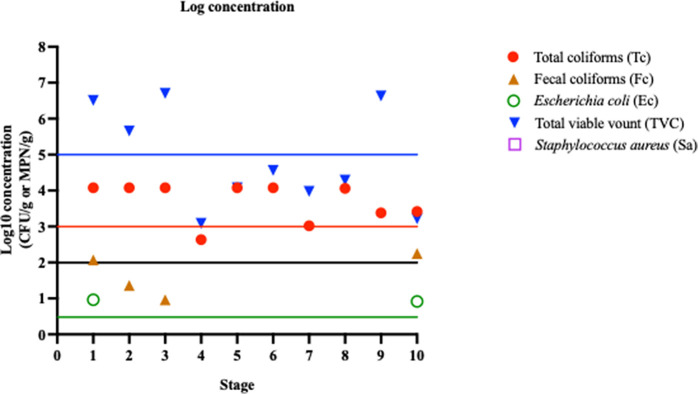
Log concentration to total variable count, total coliforms, fecal coliforms, *E. coli,* and *S. aureus* across various stages of dried shrimp production. Note: The straight line referred to the national standard of dried shrimp quality: total viable count (<10^5^ CFU/g, log_10_CFU/g = 5) (blue line); total coliforms (<10^3^ MPN/g, log_10_CFU/g = 3) (red line); fecal coliforms (<10^2^ MPN/g, log_10_CFU/g = 2) and *S. aureus* (<10^2^ CFU/g, log_10_CFU/g = 2) (black line); *E. coli* (<3 MPN/g, log_10_CFU/g ≈ 0.48) (green line).

We found a high prevalence of total coliforms in the shrimp samples from central Thailand, in particular during the early processing stages and in water samples (Fig 3). This indicates possible contamination of the aquaculture environment and during shrimp harvest and washing. Although a decreasing trend in fecal coliform and *E. coli*–positive samples was observed across the multiple processing stages, this finding should be interpreted with caution because different sample sets were analyzed at each stage. These findings underscore the essential role of the boiling and drying stages in reducing bacterial contamination levels during dried shrimp processing. However, this study also documented evidence of recontamination and/or bacterial regrowth during the packaging stages, given that 16.7% (4/24) of samples were positive for both fecal coliforms and *E. coli*, with MPN/g values exceeding the limits set by FAO (fecal coliforms ≤100 MPN/g) and TAS 7012 (*E. coli* ≤ 3 MPN/g) for each of these bacterial standards. This recontamination and/or regrowth pattern aligns with prior research reporting that hygiene lapses during the handling and packaging of dried seafood products can contribute to microbial deterioration [30].

Total viable counts fluctuated during production, with a 4-log reduction observed at the cooking stage (stage 5). However, subsequent production stages exhibited a 1–4 log increase (Fig 3). Cooking effectively reduced total and fecal coliform levels, but the final dried shrimp products often exceeded food quality standard limits. In addition, our survey revealed that shrimp are typically dried outdoors on mesh racks, wooden boards, or concrete surfaces, with most sorting and shell removal done manually. These exposed environments and handling characteristics increased the risk of bacterial recontamination and/or regrowth after cooking, especially when conducted without protective coverings or environmentally controlled drying conditions. While the survey data were descriptive and not statistically tested, these findings provides valuable insights into the factors contributing to the increase in microbial levels during later stages of production. Therefore, strengthening hygiene practices and implementation of critical control points after cooking should improve the microbial safety of dried shrimp from these regions.

In general, failure to achieve minimal salt concentrations and maximal levels of percent moisture and water activity for low moisture foods can create the conditions for microbial regrowth and reduce food safety. In our study, only a handful of the 75 final samples of dried shrimp had detectable *S. aureus* (2 samples) and *E. coli* (4 samples). Interestingly, the *S. aureus*-contaminated samples also exhibited moisture and water activity levels exceeding established limits, suggesting that inadequate drying and/or improper post-drying handling might have occurred, both of which are known to facilitate the survival or regrowth of this bacterium. Similarly, two of four *E. coli*-contaminated samples had excessive water activity levels. The measured water activity (a_w_) across all samples varied from 0.734 to 0.937. This variability in a_w_ can lead to different shelf lives for batches of dried shrimp exhibiting higher or lower a_w_, as both chemical spoilage and microbial growth are affected by a_w_. Maintaining minimal levels of a_w_ is crucial for ensuring the quality and food safety of dried shrimp, given that most bacteria require a_w_ levels above 0.61 to proliferate [29]. Good quality dried seafood typically has a pH between 6.0 and 6.9. However, sun-dried samples stored in air pouches showed a higher pH of 7.70. This increase in pH may be due to extended storage times, which can lead to a decline in product quality [30].

Although *S. enterica* serovars Tallahassee and Mount Pleasant were not detected in the final product samples, their presence in water from the sampled aquaculture environment suggests a potential source of cross-contamination that has not yet been reported previously for aquaculture settings, in contrast to the established detection of these serovars in humans, poultry, and captive animals [31–33]. This indicated that environmental monitoring may be crucial to prevent contamination of aquaculture products with *S. enterica* from non-aquatic sources.

This study highlights that while the cooking process effectively reduces bacterial loads in dried shrimp, post-processing contamination and/or regrowth and factors such as pH, production location, color, and fecal coliform levels were significantly associated with the final *E. coli* concentration in the product, often exceeding safety standards. Non-colored dried shrimp with a pH under 8, meeting the TAS standard, exhibited significantly lower *E. coli* levels compared to samples exceeding pH 8. This difference in *E. coli* levels is likely the result of higher acidic environments hindering the growth and survival of *E. coli*. Bacteria, including *E. coli*, thrive within specific pH ranges, and deviations of pH outside this range can disrupt bacterial metabolism and replication, potentially causing cell death [34]. The significant pH–color interaction observed in the multivariable model indicates that pH does not uniformly influence *E. coli* across all sample types, explaining the discrepancy between the univariate and adjusted analyses. The bacterial effect of pH was effectively nullified for colored shrimp, suggesting that the coloring pigments themselves and/or some mechanisms associated with addition of these chemicals modified how pH affects bacterial levels. This highlights the importance of considering effect modification in microbial assessments, as overlooking such interactions may mask meaningful variability among sample groups.

*E. coli* contamination levels in the dried shrimp differed across study sites, indicating the impact of varying environmental factors, including water quality, salinity, temperature, and potential fecal sources during harvesting. Based on spearman rank correlations which were not adjusted for other variables as in a multivariable model, higher relative humidity was associated with increased *E. coli* levels, and warmer, drier temperatures were associated with reduced fecal coliform counts. Other environmental parameters analyzed in this study were not significantly associated with microbial loads, suggesting that weather variability may have a limited influence on *E. coli* levels in finished dried shrimp compared to the effects of processing and hygiene practices. Nevertheless, the identified associations highlight that ambient environmental factors can shape initial contamination levels, suggesting that the Different shrimp species were collected at various production stages, resulting in discrepancies between stage samples and final dried products. These variations reflect the natural changes in species availability during production, making it difficult to trace the same species from start to finish. Different shrimp species were collected at various production stages, resulting in discrepancies between stage samples and final dried products. These variations reflect the natural changes in species availability during production, making it difficult to trace the same species from start to finish. Preferred locations to harvest shrimp would be those with consistently low bacterial contaminants in the water, proper sanitation nearby, and compliance with established water quality regulations. Dried shrimp with natural coloring showed a tendency for higher *E. coli* levels compared to those with added color, but only when levels of pH exceeded 8, otherwise there was little difference in the *E. coli* levels between colored and non-colored when pH was below 8.

To promote food safety of dried shrimp, our findings point to the need for key interventions at critical stages. Post-cooking bacterial recontamination and/or regrowth must be prevented by creating segregated, clean handling zones and implementation of rigorous personnel and equipment hygiene practices. Beyond sanitation, product quality and safety can be improved by optimizing conditions, including controlling moisture and temperature during drying, adjusting salt and pH levels, and formally implementing Critical Control Points (CCPs) that govern effective cooking, physical separation of raw and finished products, appropriate packaging, and controlled storage environments.

## Conclusions

The observed variations in the physical and chemical characteristics of retail dried shrimp highlight the importance of routine and comprehensive quality assessments. The detection of potentially pathogenic bacteria, including *E. coli* and *S. aureus*, in final products points to critical gaps in hygiene and handling practices. As the pathogenicity of those isolates was not assessed, further studies are needed to clarify their potential health risks. While boiling and drying reduce fecal coliforms and *E. coli*, the risk of re-contamination and likelihood of regrowth of low levels of these bacteria during packaging remains significant. We strongly recommend implementing strict, standardized quality control and hygiene protocols throughout all stages of production, including handling, packaging, and storage, along with regular microbial monitoring. These measures are essential to safeguard consumer health and ensure the consistent production of safe, high-quality dried shrimp.
